# Increasing European Support for Neglected Infectious Disease Research

**DOI:** 10.1016/j.csbj.2017.01.007

**Published:** 2017-01-19

**Authors:** Ole F. Olesen, Marit Ackermann

**Affiliations:** European & Developing Countries Clinical Trials Partnership (EDCTP), Anna van Saksenlaan 51, 2593 HW, The Hague, The Netherlands

## Abstract

Neglected infectious diseases (NIDs) are a persistent cause of death and disability in low-income countries. Currently available drugs and vaccines are often ineffective, costly or associated with severe side-effects. Although the scale of research on NIDs does not reflect their disease burden, there are encouraging signs that NIDs have begun to attract more political and public attention, which have translated into greater awareness and increased investments in NID research by both public and private donors. Using publicly available data, we analysed funding for NID research in the European Union's (EU's) 7th Framework Programme for Research and Technological Development (FP7), which ran from 2007 to 2013. During FP7, the EU provided €169 million for 65 NID research projects, and thereby placed itself among the top global funders of NID research. Average annual FP7 investment in NID research exceeded €24 million, triple that committed by the EU before the launch of FP7. FP7 NID projects involved research teams from 331 different institutions in 72 countries on six continents, underlining the increasingly global nature of European research activities. NID research has remained a priority in the current EU Framework Programme for research and innovation, Horizon 2020, launched in 2014. This has most notably been reflected in the second programme of the European & Developing Countries Clinical Trials Partnership (EDCTP), which provides unprecedented opportunities to advance the clinical development of new medical interventions against NIDs. Europe is thus better positioned than ever before to play a major role in the global fight against NIDs.

## 1 Introduction

Neglected infectious diseases (NIDs) comprise a highly diverse group of communicable illnesses that disproportionately affect poor populations in low- and middle-income countries (LMICs) in tropical and subtropical parts of the world. NIDs affect more than one billion people [1] and are responsible for more than 500,000 deaths every year [2]. While NIDs disproportionately affect the poorest countries in the world, they are not exclusively a problem of developing countries. The recent and devastating outbreak of Ebola in several West African countries demonstrated that NIDs can rapidly develop into a global threat. It also illustrated how social stability, economic growth, regional peace and national security can be threatened by an emerging disease outbreak when no adequate medical interventions are available.

Combating and controlling NIDs is therefore a global challenge, but a number of scientific and commercial obstacles impede the development of new medical products. Of the 850 new therapeutic products registered between 2000 and 2011, only 18 (2%) were indicated for NIDs [3]. As a result, patients with NIDs are often treated with antiquated drugs that are ineffective, toxic or difficult to administer, while for some NIDs such as Buruli ulcer no drugs or vaccines of any kind are available.

When world leaders adopted the Millennium Declaration in September 2000 [4], the fight against “HIV/AIDS, malaria and other diseases” was included as the sixth goal. This resulted in significant global support to combat the three major poverty-related diseases (HIV/AIDS, malaria and tuberculosis), whereas other important diseases of poverty were largely overlooked [5]. This situation has improved significantly over recent years, and the international community has become increasingly aware of the importance of confronting NIDs. In their statement of 9 October 2015, the ministers of science from the G7 countries expressed their resolve to support the fight against neglected tropical diseases, in line with the declaration of the G7 leaders at their meeting in Elmau, Germany on 8 June 2015 [6]. Similarly, the heads of state of the BRICS countries (Brazil, Russia, India, China and South Africa) included the fight against neglected tropical diseases in the Ufa declaration in July 2015 [7]. These high-level statements align well with target 3–3 of the Sustainable Development Goals, which promises to end the epidemics of AIDS, tuberculosis, malaria and neglected tropical diseases, and to combat hepatitis, water-borne diseases and other communicable diseases by 2030.

In parallel with this greater political attention, several major donor organisations have increased their funding of NID research. Despite the financial crisis, overall global R&D funding for 13 neglected tropical diseases increased by more than 70% between 2007 and 2011, from US$268 million to US$464 million [8]. This has partly been a consequence of increased donations from private charitable organisations such as the Bill and Melinda Gates Foundation, but LMICs, in particular some emerging economies, have also begun to support NID research on a larger scale. More academic and public research organisations have therefore increased their engagement in NID research, while a number of pharmaceutical companies have created dedicated facilities for the development of new interventions against NIDs.

The largest funder of public research within Europe is the European Union's (EU's) multiannual Framework Programme (FP), which has supported research on NIDs since the 4th Framework Programme (FP4, 1994–98), when International Cooperation (INCO) activities were introduced. INCO was one of the first major research funding schemes to focus specifically on NID research through transnational collaborative research. Between 1997 and 2006, it provided some €70 million support for 55 NID research projects. The projects covered a wide range of research areas, from vector control to vaccine research, as well as development of non-medical innovations such as traps for tsetse flies and solar-powered disinfection of drinking water. The projects included research on individual diseases but also addressed complex diseases such as childhood infections and diarrhoeal diseases, as well as research on health systems and health service issues of disease control.

Building on the activities of the INCO programme, NID research was identified as a specific priority for the 7th EU Framework Programme (FP7; 2007–13) [9]. FP7 had a total indicative budget of more than €50 billion and was composed of four major sub-programmes (in addition to a special sub-programme on nuclear research). The largest sub-programme, Cooperation, represented two-thirds of the overall budget and focused on collaborative research between research teams in different countries. While research teams from most countries in the world could participate and receive funding from the Cooperation sub-programme, individual projects should always include organisations from at least three different EU or FP7-associated countries, thereby giving a bias towards participation and funding of European institutions. The Ideas programme, with the European Research Council (ERC) as its flagship initiative, supported individual research teams around a principal investigator. The People programme, including Marie Curie actions, provided fellowships for researcher mobility and career development. The Capacities programme, the smallest of the four sub-programmes, was predominantly aimed at strengthening research infrastructure.

Using publicly available information, we have analysed FP7 funding for NID research, to determine whether European support in this area has matched the global increase in funding. We also analysed the specific diseases and pathogens targeted in FP7 NID projects and the type of research funded.

## 2 Methods

We used the European Commission's Community Research and Development Information Service (CORDIS, cordis.europa.eu) to identify NID research projects funded during FP7 (2007–13). Search terms included broad classifications of pathogens (e.g. helminth, kinetoplastid, protozoa, virus, worm) and diseases (e.g. diarrhoea, filariasis, neglected infectious diseases, neglected tropical disease, trypanosomiasis) as well as specific infections (e.g. dengue, elephantiasis, leishmaniasis, rabies, schistosomiasis) and organisms (e.g. bancrofti, buruli, cruzi, leprae, shigella). This resulted in a gross list of 640 projects of potential interest. The abstracts of these projects were then examined to identify projects for which NID research was the core activity, resulting in a shortlist of 65 projects. These projects were subsequently categorised according to the diseases and pathogens they addressed, and according to the type of research funded. A few projects were addressing both NID and other diseases, in which case they were proportioned evenly between NID and other diseases. Financial data and information about participants in the shortlisted projects were obtained from the CORDIS database and cross-checked with downloaded data from the EU Open Data Portal (https://open-data.europa.eu/en/data/dataset/cordisfp7projects). For collaborative projects involving multiple partners, the distribution of project budgets among partners was available in most cases through the Open Data Portal. However, for a few projects (approximately 5%) only the total project budget was available and in these cases we assumed that the total budget was distributed evenly among all project partners.

To compare the funding for NID research from major funding organisations, we used the G-Finder Public Search Tool (https://gfinder.policycures.org/PublicSearchTool/searchDisease). For each funder we extracted the total disbursements in the period from 2007 to 2014 for all of the 35 diseases covered by the G-Finder database and subsequently subtracted disbursements for HIV/AIDS, malaria, tuberculosis and non-allocated research to arrive at an estimated level of funding for NID research.

## 3 Results

**Fig. 1:**
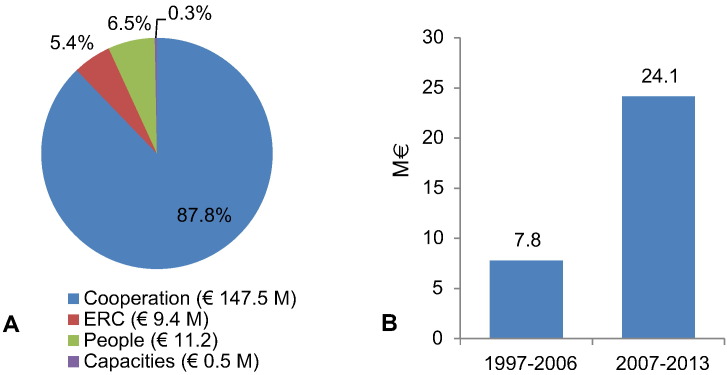
A: The total financial contribution in FP7 to NID research comprised €168.2 M, with the majority of funding coming from the Cooperation sub-programme; B: The average annual contribution to NID research from the EU framework programmes during the periods of 1997–2006 and 2007–2013.

We identified 65 NID research projects that were funded during the seven years of FP7 and received a total financial contribution of almost €169 million (Fig. 1). This corresponds to an average annual commitment of more than €24 million. By comparison, less than €8 million was disbursed on average during the nine years of the INCO programme. EU funding for NID research therefore tripled in the period 2007–13 in comparison to the preceding decade. NID research was funded within each of the four major sub-programmes of FP7, but more than 87% of funding (€147.5 million) was allocated to just 34 projects within the Cooperation programme.

Kinetoplastid diseases (leishmaniasis, trypanosomiasis and Chagas disease) received €67 million, making it the largest disease area in terms of EU funding (Table 1). One of the kinetoplastid diseases, leishmaniasis, received the most funding dedicated to a single disease, €20.5 million. With an EU contribution of €31 million to 10 projects, research on helminth diseases was the second largest funding area; around half of the projects (five projects, €9.3 million) targeted schistosomiasis. Some €30 million was allocated to 17 projects on bacterial diseases, with more than three-quarters of this funding (€22 million) being allocated to research on diarrhoeal diseases. Finally, almost €23 million was allocated to three cross-cutting research projects spanning more than one group of pathogens.

More than three-quarters of EU support for research on NIDs (€121.7 million) was invested in projects that could be classified as product development of new drugs, vaccines and diagnostics (Table 2). Just 5% of funding (€9.4 million) was invested in basic research activities, mainly through ERC grants and Marie Curie actions. Vaccines constituted the largest focus area across diseases, with a total funding of more than €61 million. All projects included various elements of capacity building, but projects with a dedicated focus on research training and strengthening of research infrastructure in endemic regions received €13.1 million.

A more detailed analysis revealed that 481 research teams were involved in EU-funded NID research projects. Some 67% of these 481 research teams were from academic (212) or public (110) research institutions, whereas participation from the private sector was more evenly distributed between non-profit organisations (63) and for-profit companies (75). In some cases, the same research team participated in two or more FP7 projects, while some institutions participated with different research teams in separate FP7 projects. The participating research teams nevertheless represented 325 different organisations from 72 countries on six continents. Out of these, 65 institutions (20%) were based in 38 LMICs in Africa (20), Asia (8) and Latin America (10). While these institutions represented about a fifth of all participants, they collectively received less than €25 million (15%) of the total funding.

We subsequently examined how research funding was distributed among the participating organisations and identified a large variation in the amount of funding received by different organisations. The 10 largest recipients of FP7 funding for NID research included many of Europe's largest and most prestigious institutes for tropical and infectious diseases, such as the Pasteur Institute in Paris, the London School of Hygiene and Tropical Medicine, the Swiss Tropical and Public Health Institute, and the Belgian Institute of Tropical Medicine in Antwerp (Table 3). Collectively, these top ten institutions received more than 20% of all FP7 funding for NID research.

To estimate how the level of EU funding for NID research compares with other funders of global health we used the G-Finder Public Search Tool, which contains information on global disbursements for research into 35 neglected diseases. The group of diseases in the G-Finder survey is slightly different from the group of diseases in our study. In addition, the G-Finder data are based on actual or estimated disbursements in a given year, rather than total investments over the lifetime of a project or an activity. Despite these shortcomings, the G-Finder data base can nevertheless provide a fair estimate of the level of funding from different funders. We found that the US National Institutes of Health (NIH) and the Bill and Melinda Gates Foundation (BMGF) were by far the largest global funders of NID research. In the period from 2007 to 2014, the total disbursements to disease-specific NID research (excluding disbursements to HIV/AIDS, malaria and tuberculosis) from these two organisations amounted to more than 1.8 billion and 1.0 billion USD, respectively. The third largest funder of NID research was the Wellcome Trust with disbursements of 237 million USD, while the European Commission took fourth place with 166 million USD. This was followed by two French organisations, INSERM and the Pasteur Institute in Paris, which both invested slightly more than 110 million USD, while the US Department of Defence (DoD) disbursed approximately 100 million USD.

## 4 Discussion

A total of €169 million was spent by the EU on NID research during the 7 years of FP7, targeting 65 research projects. This is more than the EU investments in malaria (€122 million) and tuberculosis (€118 million), and comparable to the EU investments in HIV/AIDS (€175 million) in the same period [10]. However, the total budget for the health research programme in FP7 was more than €6.1 billion, and NID research therefore received less than 3% of all health research investments.

Global support for NID research increased by approximately 70% from 2007 to 2011 [8], but our analysis demonstrates that average annual financial support for NID research from the EU increased by 200% during the implementation of FP7. European support for NID research therefore grew almost three times faster than the global average during FP7. It should be noted however that the increased support for NID was associated with a concurrent decrease in support for the three major poverty-related diseases (HIV/AIDS, malaria and tuberculosis). The total EU contribution to research activities for the big three in the four years of FP6 (2002–2006) was thus more than €455 million (including a contribution of €200 million to the first phase of the EDCTP programme), corresponding to approximately 119 million per year [11]. In comparison, the annual average research investments for HIV/AIDS, malaria and tuberculosis was less than €60 million during the seven years of FP7.

In the period from 2007 to 2014, the EU was one of the world's largest funders of NID research, together with the US National Institutes of Health, the Bill and Melinda Gates Foundation and Wellcome Trust [12]. It is notable that the largest European investments have been made in vaccine research, while many other major funders have focused primarily on the development of new or improved drugs for NIDs. The strong focus on vaccine research can probably not be explained by a single factor, but an important element could be the strong historical legacy and industrial base for vaccine research in Europe.

The increased investments in NID research in Europe and globally have generated an increasing number of candidate products. The product pipeline has more than doubled in size over the last decade, and currently comprises more than 167 candidates for new drugs, vaccines and diagnostics [13].

Several of these candidates should be advanced over the coming years. However, bringing new products through clinical development and to market requires substantial financial resources. The shortage of human and institutional capacity to perform clinical trials to international standards in disease-endemic countries is another important bottleneck. The principal European response to address these challenges has been an expansion of the European & Developing Countries Clinical Trials Partnership (EDCTP) – a pooled funding mechanism jointly owned by a group of African and European countries and financially supported by the EU. Currently, EDCTP has 28 participating countries, 14 European and 14 from sub-Saharan Africa. It was established in 2003 as a response to the Millennium Development Goals, and had the objective of accelerating the clinical development of new medical products against poverty-related diseases. Since its establishment, EDCTP has funded more than 240 projects, and contributed to 102 clinical trials, while also supporting local capacity strengthening through fellowships, networking and infrastructure development.

The second phase of EDCTP (EDCTP2) was launched in December 2014 with a financial contribution of up to €683 million from the EU's Horizon 2020 Framework Programme, the successor to FP7 [14]. EU contributions will match funds provided by participating European governments, either in cash or in-kind, while additional contributions and collaborations will be sought from African governments, industry, private charity funders and like-minded organisations. Taken together, the EDCTP2 programme has therefore a projected financial volume of more than €1.8 billion over a 10-year period, making it the world's largest pooled funding mechanism with a specific focus on poverty-related disease research.

While the first EDCTP programme focused on HIV/AIDS, malaria and tuberculosis, EDCTP2 has a wider remit that includes NIDs. The additional funding will enable EDCTP2 to support all phases of clinical trials (phases I–IV) and it can therefore fill an important gap in the development, clinical evaluation and uptake of new products for NIDs. EDCTP2 also aims to ameliorate the shortage of human and institutional capacity for NID research. Collaborative research is a cornerstone of EDCTP2, as the larger clinical trials must be carried out in partnership between research teams from Europe and sub-Saharan Africa. This will help to create multidisciplinary consortia and networks of NID scientists spanning multiple institutions and countries, generating a critical mass with the expertise and capacity to undertake complex clinical trials in resource-poor settings.

The focus of FP7 on translational and collaborative research consortia has undoubtedly strengthened the capacity of European scientists to discover and develop new products against NIDs in collaboration with disease-endemic countries. European-funded research progressed a number of vaccines, drugs and diagnostic tests from preclinical testing into later development stages. Vaccine and drug candidates against leishmaniasis, helminths and diarrhoea have been moved from pre-clinical to early clinical trials, while other projects strengthened understanding of co-infections between poverty-related diseases and helminth infections, and discovered new biomarkers and genetic factors relevant to disease progression and drug resistance. With the expanded remit of EDCTP2, promising candidate products can be progressed through the development pipeline. EDCTP2 can also catalyse their successful uptake by the healthcare systems by supporting, in collaboration with other partners, important public health aspects like health education, pharmacovigilance, and cross sectoral approaches (One Health) to combat neglected zoonoses. At the same time it is important to continue strengthening the capacity for basic research in disease-endemic countries in order to create a critical mass of expertise that could have a long-lasting effect on the scientific landscape. The strong focus on collaborative research in FP7 has strengthened the links between research teams in different countries, including disease-endemic countries, and helped to create a global network of NID researchers. This network forms a solid basis for further collaborative research that could have a lasting impact on human health and progress towards the Sustainable Development Goals.

## Figures and Tables

**Table 1:** FP7 Funding of NID research: number of projects and EU contribution per disease.

Disease class	Disease	Number of projects	EU contribution (€ million)
Protozoan	Chagas disease	5	7.49
	Leishmaniasis	9	20.46
	Trypanosomiasis	3	4.35
	Multiple protozoan diseases	11	34.88
Helminth	Cystic echinococcosis	1	2.86
	Hookworm	1	6.00
	Onchocerciasis	1	5.00
	Schistosomiasis	5	9.31
	Multiple helminth diseases	2	7.64
Bacterial	Borreliosis	1	3.00
	Buruli ulcer	2	4.76
	Cholera	4	2.12
	*Clostridium difficile* disease	1	0.53
	Shigellosis	5	2.85
	Multiple pathogen diarrhoea	3	16.62
	Multiple bacterial diseases	1	0.05
Viral	Dengue fever	1	6.00
	Rabies	1	2.99
	West Nile fever	3	3.09
	Multiple viral diseases	2	5.65
Multiple	Multiple diseases	3	22.98
Total		65	168.62

**Table 2:** FP7 Funding of NID Research: Funding per research activity (€ million).

	Bacterial diseases	Helminth diseases	Protozoan diseases	Viral diseases	Multiple diseases	Total
Basic research	5.2	2.6	1.6	0	0	**9.4**
Drug development	0.5	3.3	41.8	3.0	0	**48.6**
Vaccine development	24.2	19.9	11.4	5.7	0	**61.3**
Diagnostic tools	0	2.9	4.0	0	5.0	**11.8**
Vector control	0	0	0.2	0.1	12.0	**12.3**
Research training	0	0	3.8	0	0	**3.8**
Research infrastructure	0	0.4	0	2.9	6.0	**9.3**
Other	0	1.7	4.4	6.0	0	**12.1**
Total	**29.9**	**30.8**	**67.2**	**17.7**	**23.0**	**168.6**

**Table 3:** Top 10 Institutions receiving FP7 funding for NID research.

Participating institution	Location	Number of projects	FP7 funding (€ million)
Pasteur Institute, Paris	France	12	8.8
Swiss Tropical and Public Health Institute, Basel	Switzerland	7	3.9
University of Edinburgh	UK	4	4.7
London School of Hygiene and Tropical Medicine	UK	6	3.1
Institute of Tropical Medicine, Antwerp	Belgium	6	2.8
University of Oxford	UK	5	2.9
Queen's University Belfast	UK	1	2.5
Leiden University Medical Center	Netherlands	4	2.7
Spanish National Research Council (CSIC)	Spain	7	2.6
Oswaldo Cruz Foundation (Fiocruz)	Brazil	6	2.2
